# From Semantics to Feelings: How Do Individuals with Schizophrenia Rate the Emotional Valence of Words?

**DOI:** 10.1155/2012/431823

**Published:** 2012-06-06

**Authors:** Ana P. Pinheiro, Robert W. McCarley, Elizabeth Thompson, Óscar F. Gonçalves, Margaret Niznikiewicz

**Affiliations:** ^1^Neuropsychophysiology Lab, CIPsi, School of Psychology, University of Minho, 4710-057 Braga, Portugal; ^2^Clinical Neuroscience Division, Laboratory of Neuroscience, Department of Psychiatry, Boston VA Healthcare System, Brockton Division and Harvard Medical School, Boston, MA 02301, USA

## Abstract

Schizophrenia is characterized by both emotional and language abnormalities. However, in spite of reports of preserved evaluation of valence of affective stimuli, such as pictures, it is less clear how individuals with schizophrenia assess verbal material with emotional valence, for example, the overall unpleasantness/displeasure relative to pleasantness/attraction of a word. This study aimed to investigate how schizophrenic individuals rate the emotional valence of adjectives, when compared with a group of healthy controls. One hundred and eighty-four adjectives differing in valence were presented. These adjectives were previously categorized as “neutral,” “positive” (pleasant), or “negative” (unpleasant) by five judges not participating in the current experiment. Adjectives from the three categories were matched on word length, frequency, and familiarity. Sixteen individuals with schizophrenia diagnosis and seventeen healthy controls were asked to rate the valence of each word, by using a computerized version of the Self-Assessment Manikin (Bradley and Lang, 1994). Results demonstrated similar ratings of emotional valence of words, suggesting a similar representation of affective knowledge in schizophrenia, at least in terms of the valence dimension.

## 1. Introduction

Emotional abnormalities are a hallmark of schizophrenia [1–4] and are often evident in prodromal stages of this disorder [1, 5].

Recent years have seen a rapid increase in interest in emotion processing in schizophrenia. Stimuli with emotional salience have particular relevance for the individual and, thus, abnormalities in their processing have important consequences for social functioning and functional outcomes for individuals with schizophrenia [6]. The existing studies have explored different aspects of emotion processing in this disorder (see [7] for a review) including the study of (a) emotional perception (e.g., [8]); (b) emotional experience (e.g., assessment of self-reported affect through the presentation of emotionally evocative stimuli; assessment of trait differences in emotion components) (e.g., [9]); (c) emotional expression [10]; (d) effects of emotion on cognitive processes, such as working memory [11, 12]; (e) evaluation of the affective properties of stimuli varying in valence and arousal. In terms of the conceptual framework, the latter studies represent a dimensional approach to emotion. Dimensional theories of emotion propose that emotions can be characterized along a small number of underlying and separable dimensions, such as valence (the overall unpleasantness/displeasure relative to pleasantness/attraction of a stimulus) and arousal (the intensity of motivational mobilization—appetitive or defensive) [13–15]. This assumption is supported by brain functional imaging (e.g., [16]) and event-related potential (ERP) studies (e.g., [17]) indicating differential effects of valence and arousal on brain activation and function. In contrast, a discrete emotions approach holds that emotions may be distinguished from one another according to a set of features [18].

The existing studies on emotion processing in schizophrenia suggest that the components of emotional processing mentioned above are differentially affected by the disorder (see [7] for a review). For example, previous studies pointed to a dissociation between the subjective experience and the expression of emotion in schizophrenia [19], based on somewhat paradoxical findings revealing that these individuals show less emotional expression, even though they report momentary emotional experience similar to that of healthy controls (HC) in response to stimuli such as film clips, pictures, or emotional face expressions (e.g., [20, 21]). Studies using self-report measures of emotional experience have additionally demonstrated that individuals with schizophrenia report experiencing feelings in a way that is consistent with the valence of the presented evocative stimuli, that is, they report negative mood states in response to unpleasant stimuli or positive mood states in response to pleasant evocative stimuli ([22–27]; see also [28] for a review). Nonetheless, other studies found that individuals with schizophrenia diagnosis report experiencing less positive emotion in response to happy emotional face expressions, in comparison with HC [25].

In particular, most studies investigating the way schizophrenic individuals rate affective properties of stimuli have provided evidence for similar evaluation of valence of affective stimuli in schizophrenic patients and HC (pictures [4, 20, 23, 29–31], facial expressions [32] and odors [33, 34]). However, these results have not been always replicated. For example, some studies indicated that schizophrenic individuals tend to rate pleasant stimuli as being less pleasant [35, 36], and negative stimuli as being less unpleasant [35, 36] (in both studies, the stimuli used were emotional pictures, words, and faces). Differences in patients' samples (e.g., gender [29]), including schizophrenia subtype, clinical symptoms (e.g., severity of negative symptoms and level of anhedonia) and functional outcome measures [36–38]) as well as differences in stimuli (e.g., level of arousal) may account for the apparent discrepancies between studies.

Findings related to the assessment of arousal indicate either similarities [23] or differences [21, 36, 39] in the assessment of this dimension. For example, differences were observed in arousal ratings of aversive/unpleasant stimuli, with lower ratings indicated by individuals with schizophrenia relative to HC in response to different types of stimuli, such as pictures selected from the International Affective Pictures System dataset (IAPS [39]), words selected from the Affective Norms for English Words dataset (ANEW [40]), sounds selected from the International Affective Digitized Sounds dataset (IADS [41]), or emotional faces [35]. When compared with patients with bipolar disorder and HC, schizophrenia patients reported lower arousal for aversive stimuli with social content [39]. Also, heightened arousal ratings were found for pleasant pictures [21] and for neutral stimuli (pictures, words, and faces [36]). Discrepancies in these findings might be related to methodological differences, including sample differences (e.g., differences in anhedonia level or in neurocognitive measures such as working memory [35]). In spite of differences in ratings of valence and arousal in some of individuals with schizophrenia, the existing studies point to a representation of affective knowledge in schizophrenia similar to that found in HC, suggesting that valence and arousal are also two major features of this knowledge (see also [42]).

Besides affective abnormalities, disturbance of language processes has long been reported in schizophrenia. It includes deficits in declarative-episodic memory of verbal material [43], abnormal semantic priming effects [44, 45], and abnormal context processing [46]. Abnormalities were also found in the brain network involved in semantic processing [47]. Language abnormalities in schizophrenia were proposed to rely both on an initial overly activated semantic network and on later inhibition difficulties indicating abnormal context utilization [48, 49]. These semantic processing deficits do not seem to be dependent on grammatical category of a word, such as nouns, verbs, or adjectives [50]. However, it is less clear how individuals with schizophrenia process verbal material with emotional valence.

Studies with healthy populations have demonstrated a differential processing of neutral, pleasant and unpleasant verbal information [51–56] as well as an automatic processing of emotional word content in the sense that it is not dependent on the availability of attentional resources [57]. For example, pleasant adjectives tend to be better remembered than unpleasant or neutral adjectives, suggesting a preferential processing of pleasant words [55, 57]. Electrophysiologically, the prioritized processing of emotional verbal material is indexed by enhanced (i.e., more positive or more negative) ERP amplitude for emotional relative to neutral words [54, 57, 58].

Studies testing affective semantic priming (a variant of the semantic-priming paradigm consisting of the presentation of an emotional prime word before a target word with emotional meaning) reported similar affective and semantic priming in individuals with schizophrenia when compared with HC [59, 60]. However, other studies revealed that these individuals tend to show a facilitatory priming effect for neutral word stimuli, but not for positive or negative word stimuli; in addition, schizophrenic individuals' reaction times tended to be slower for related negative word targets than to unrelated negative word targets [45].

Studies on sentence processing with affective semantic content showed abnormalities in the interaction between semantic networks and emotional processing in schizophrenia [61], as indexed by increased N400 for negative word endings relative to both depressed and HC groups. In addition, individuals with schizophrenia did not show memory enhancement for self-referenced adjectives, contrary to HC, which may be related to poor social outcomes in this disorder [62]. Interestingly, phenomenological studies on auditory verbal hallucinations show that these often have negative/derogatory semantic content [63–65], which may suggest a relationship between clinical symptoms and processing of verbal material with negative emotional valence.

In spite of evidence suggesting abnormalities in processing verbal affective stimuli, it is yet not clear if abnormalities are related to abnormal declarative knowledge about affect. A previous study [42] provided evidence for similar knowledge representations of verbal affective stimuli in 11 individuals with schizophrenia and 7 HC, in terms of their valence-based and arousal-based meaning. Differences were found in the weighting of valence and arousal dimensions in a task of similarity assessment of affective word pairs: while participants with schizophrenia weighted the valence and arousal dimensions equally, HC weighted more the arousal than the valence dimension, suggesting that the relative importance of these dimensions may differ in individuals with schizophrenia and HC. However, in this study only 16 emotion terms were used (excited, lively, cheerful, pleased, calm, relaxed, idle, still, dulled, bored, unhappy, disappointed, nervous, fearful, alert, and aroused), and they were assessed on a 7-point Likert scale (1: extremely dissimilar; 7: extremely similar). Also, Burbridge and Barch [35] assessed emotional experience to pleasant, neutral and unpleasant stimuli in different modalities, including 75 words selected from the ANEW dataset [66], varying in valence (pleasant, unpleasant, and neutral) and arousal (low and high). Schizophrenic participants and HC were asked to rate their emotional experience to the stimuli, in terms of how pleasant-unpleasant and aroused-calm the stimuli made them feel. However, in this study a composite index was used that did not allow to investigate the separate processing of word stimuli with emotional valence.

In this study, we compared valence ratings of adjectives in individuals with schizophrenia and in HC. To our knowledge, only one previous study [42] has directly assessed how individuals with schizophrenia assess emotional adjectives, in spite of substantial research on how they assess other types of affective stimuli such as pictures or film clips [4, 21, 23, 31, 67, 68]. However, the study of Kring et al. [42] included a small number of adjectives that may not be representative of the vocabulary that depicts emotional situations in the daily life.

In our study, we have presented a list of 184 adjectives (previously assessed as “pleasant,” “unpleasant,” or “neutral” by a group of 5 judges) to a group of 16 individuals with schizophrenia diagnosis and to 17 HC. They were asked to assess the valence of the words on a 1–9 Likert scale [13]. We posited that individuals with schizophrenia and HC would show similar ratings of valence of adjectives, consistent with reports of preserved assessment of affective properties of stimuli differing in valence and similar representation of emotion in schizophrenia.

## 2. Materials and Methods

### 2.1. Participants

Sixteen subjects diagnosed with schizophrenia (APA, 2002) and seventeen HC participated in the study (see Table 1). Inclusion criteria were (a) age between 18 and 50 years; (b) no history of neurological illness or traumatic head injury, defined as loss of consciousness for more than 5 minutes and/or structural sequelae following head trauma; (c) no history of alcohol or drug dependence in the past five years or abuse within the last year (DSM-IV-TR; APA-2002) with diagnoses determined by the Structured Clinical Interview for DSM-IV-TR (SCID) administration [69, 70]; (d) no hearing, vision, or upper body impairment; (e) estimated intelligence quotient (IQ) of above 80 [71]; (f) no alcohol use in the 24 hours before testing; (g) an ability and desire to participate in the experimental procedure, as demonstrated by given written informed consent, following Harvard Medical School (HMS) and Veterans Affairs Boston Healthcare System (VABHS) guidelines.

 HC subjects were recruited from Internet and newspaper advertisements and matched to the patients on the basis of age, gender, parental socioeconomic status, and handedness (Table 1). For HC, additional inclusion criteria were no history of Axes I or II disorders as determined by SCID interview [69, 70]; no history of Axis I disorder in first or second degree family members, determined by the Family History-Research Diagnostic Criteria (FH-RDC) instrument [72]; no history of attention deficit disorder, learning disability or developmental disorder, and no history of birth complications with resulting consequences for central nervous system as determined by neurodevelopmental interview [73].

The experiment was explained to each participant and all participants gave a written informed consent following HMS and VABHC guidelines. All were paid for their participation in the study.

### 2.2. Stimuli

Stimuli were 184 adjectives (see Table 3) differing in emotional valence. First, a list of neutral and emotional (pleasant or positive; unpleasant or negative) adjectives was created. Given that the desired number of adjectives for each valence type could not be found in the ANEW dataset [40], we turned to published studies that have used emotional adjectives as stimuli. Thus, we have combined words taken from the ANEW with words from those original studies that published the lists of words as supplementary material [40, 74, 75] to arrive at the final set of stimuli. Five judges (mean age ± SD =  31.4 ± 12.10 years, 3 females), all with college degree (mean years of formal education = 16), involved in research and who did not participate in the experimental task, categorized each word as “neutral,” “positive,” and “negative”: 60 words were categorized as neutral, 60 words were categorized as positive, and 64 words were categorized as negative. Neutral adjectives described less arousing and less salient traits and states (e.g., “neutral,” “blue,” and “narrow”), while positive (e.g., “brilliant,” “famous,” “elegant”) and negative (e.g., “dead,” “fearful,” “sad”) adjectives described more arousing and salient affective traits and states (see also [53]).

Psycholinguistic properties were taken from the University of Western Australia database (http://www.psych.rl.ac.uk/MRC_Psych_Db.html). Words in three valence categories (neutral, positive, and negative) were matched for number of letters (four to seven letters), and number of syllables (one to three syllables). No difference was observed between the three valence types (*P* > 0.05). Word frequency (Kucera-Francis frequency [76]) was in the range of 1–300 per million words. However, it was somewhat lower for negative adjectives relative to both positive (*P* = 0.020) and neutral (*P* = 0.002) ones (see Table 2). Familiarity, concreteness, and imageability were also lower for negative relative to both positive (*P* < 0.001) and neutral (*P* < 0.001) adjectives.

### 2.3. Procedure

Adjectives were pseudorandomized and presented to each participant. Pseudorandomization was used in order to avoid sequential presentation of more than 5 stimuli with similar emotional valence. Words were presented in lowercase in 6 blocks in the center of a CRT computer screen, in black Arial 40-point font against white background. Each block consisted of 30 words. A short pause (self-paced) followed each block to minimize participants' fatigue or distraction. Before the presentation of a word, the fixation cross (5000 ms of duration) appeared. A word was then presented for 3000 ms. Following the word presentation, participants had 15000 ms to respond. They were instructed to read each word silently and to rate its emotional valence by using a computerized version of the Self-Assessment Manikin [13]. In this system, each affective dimension is assessed on a 1–9 Likert scale: higher numbers in the valence dimension indicate evaluation as more pleasant. Mean item ratings less than 4 were classified as unpleasant, between 4 and 6 were classified as neutral, and greater than 6 were classified as pleasant. Participants were also given a chance of correcting their response, in case they felt they made a mistake when pressing a button. After the response, a slide appeared (with no time limit) asking participants to confirm (by pressing button 1) or to correct their response (by pressing button 0). If subjects wanted to correct it, the trial would restart. After the confirmation of the response, an interstimulus interval (ISI) of 1000 ms preceded the onset of the next trial. 

The rating session was preceded by a practice session. Subjects were given detailed instructions and presented with a block of 9 selected words that were not shown during the actual experiment. All stimuli were presented and synchronized through SuperLab 4.2 (Cedrus Corporation, San Pedro, CA, USA). The same software was used for recording subjects' responses. Data were analyzed with IBM SPSS Statistics 19 (IBM Corporation, Armonk, NY, USA).

## 3. Results and Discussion

Words from different categories were rated differently, as suggested by the significant effect of valence (*F*(2,30) = 182.06, *P* < 0.001). No group effect or interaction involving group factor were observed. Positive words were rated as higher in valence, followed by neutral, and finally by negative words (*P* < 0.001 for all comparisons).

However, independent *t*-tests examining group differences for particular items showed differences in ratings for a subset of words. In general, some of the words that were previously categorized as “neutral” by a group of volunteers were rated more positively by individuals with schizophrenia when compared with HC, such as “blue” (*P* = 0.013; SZ = 6.75; HC = 5.18); “basic” (*P* = 0.036; SZ = 6.25; HC = 5.29); “common” (*P* = 0.026; SZ = 6.20; HC = 5.29); “central” (*P* = 0.025; SZ = 6.13; HC = 5.24); “daily” (*P* = 0.020; SZ = 6.75; HC = 5.53); “plural” (*P* = 0.049; SZ = 5.63; HC = 5.00)

 In addition, the word “genial,” previously categorized as “positive” by a group of volunteers, was rated was rated less positively by individuals with schizophrenia relative to HC (*P* = 0.001; HC = 6.88; SZ = 4.81).

Finally, some of the words that were previously categorized as “negative” by a group of volunteers were rated less negatively by individuals with schizophrenia when compared with HC: “dreadful” (*P* = 0.030; HC = 2.00; SZ = 3.31); “dead” (*P* = 0.046; HC = 1.29; SZ = 2.38); “furious” (*P* = 0.033; HC = 2.12; SZ = 3.44); “guilty” (*P* = 0.031; HC = 2.00; SZ = 3.00); “shamed” (*P* = 0.038; HC = 2.18; SZ = 3.38); “selfish” (*P* = 0.026; HC = 2.00; SZ = 2.94); “ugly” (*P* = 0.052; HC = 2.35; SZ = 3.56).

Given that the words “genial,” “furious,” and “selfish” have lower frequency values than the other words for which the groups' ratings differed (Kucera-Francis frequency = 5, 8, and 8, resp.), we have tested for the effects of years of education on differences in valence ratings of these specific words. Therefore, we have included education as a covariate in our ANOVA. No significant effect was found (“genial;”  *P* = 0.131; “furious;” *P* = 0.937; “selfish;” *P* = 0.423).

**Figure 1:**
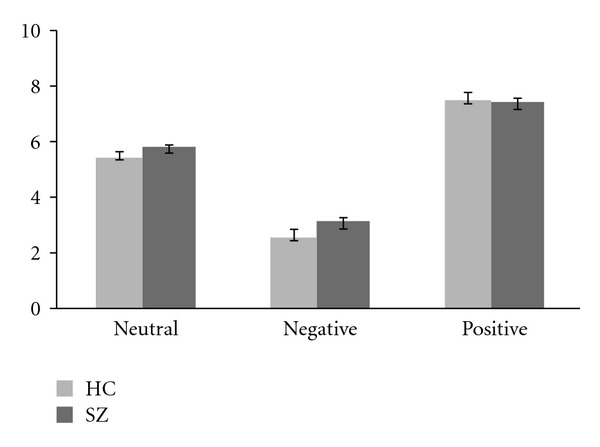
Ratings of adjectives previously categorized as “neutral,” “positive,” and “negative” by healthy controls and schizophrenic individuals.

These findings are consistent with previous studies reporting similar evaluation of valence of affective stimuli in individuals with schizophrenia and HC (e.g., [4, 9, 20, 21, 31]), supporting the hypothesis that the representation of emotion in schizophrenia is similar to controls (at least in terms of the valence dimension) (Figure 1).

However, the current results should be considered in the context of such limitations, as the small sample size and the fact that schizophrenic individuals were medicated. A larger sample including more women with schizophrenia diagnosis will allow, as well, the investigation of potential gender differences in the representation of affective knowledge for verbal stimuli [77, 78]. Also, given that findings on emotional experience (that include ratings of stimuli with affective properties) are more variable than findings on emotional expression, possibly due to differences in the stimuli used (e.g., face expressions, pictures, odors) and to differences in patients' samples (e.g., schizophrenia subtype, gender, clinical symptoms), replication of these findings is needed with different schizophrenia subtypes and clinical symptoms (e.g., positive versus negative symptomatology) (see [7] for a review of emotional response deficits in schizophrenia).

Future studies could extend the current findings by exploring how schizophrenic individuals assess the arousal of affective verbal material. This could be done by incorporating a 1–9 scale for arousal ratings (from not arousing to extremely arousing) as suggested by Bradley and Lang [13], allowing for the study of group differences in the intensity of stimuli.

Additionally, since deficits in emotion processing are already observed in the prodromal stage of the disorder [5], it would be interesting to explore the representation of affective knowledge and its effects on processing of verbal affect-related stimuli in prodromal and first-episode schizophrenic individuals in comparison with HC and chronic schizophrenia. This would allow a better understanding of possible changes in emotional processing before the frank onset of psychosis and in the first stages of the disease, particularly in terms of ratings of stimuli's affective properties. Future studies should address these issues and questions.

## 4. Conclusions

This study aimed to investigate how schizophrenic individuals rate the valence of adjectives, when compared with healthy controls. Results indicated similar ratings of emotional valence of words, providing support for a similar representation of affective knowledge related to words in schizophrenia, at least in terms of the valence dimension. Therefore, these findings further suggest that the process of extracting emotional information from semantically emotional words is similar in individuals with schizophrenia and healthy controls, increasing the confidence in self-reports of affect in this clinical group.

## Figures and Tables

**Table 1:** Demographic and clinical characteristics of participants (mean ± SD).

Variable	Healthy controls (*n* = 17)	Individuals with schizophrenia (*n* = 16)	*t *(df = 31)*	*P* value
Age (years)	43.65 ± 11.32	48.69 ± 8.38	−1.446	.158
Gender	3 females; 14 males	4 females; 12 males	NA	NA
Education (years)	15.47 ± 1.81	13.00 ± 2.25	3.487	.001
Subject's SES	2.23 ± 1.01	3.63 1.41	−2.992	.006
Parental SES	2.54 ± 0.88	3.13 ± 1.45	−1.275	.213
Onset age (years)	NA	30.23 ± 11.95	NA	NA
Duration (years)	NA	17.15 ± 11.32	NA	NA
Chlorpromazine equivalent (mg)	NA	381.13 ± 247.18	NA	NA
Medication type	NA	First generation = 3 Second generation = 9	NA	NA
Positive scale PANSS	NA	23.87 ± 9.41	NA	NA
Negative scale PANSS	NA	22.33 ± 9.56	NA	NA
General psychopathology PANSS	NA	43.00 ± 14.64	NA	NA
Total psychopathology PANSS	NA	89.20 ± 30.17	NA	NA

SES: socioeconomic status; NA: not applicable; *Independent sample *t*-test was used for group comparisons.

**Table 2:** Psycholinguistic properties of adjectives used in the experiment.

Psycholinguistic	Adjectives' valence		
measure	Neutral (*M* ± SD)	Positive (*M* ± SD)	Negative (*M * ± SD)
Kucera-Francis written frequency	114.93 ± 141.98	96.92 ± 163.10	41.39 ± 51.42
Familiarity	561.24 ± 44.44	568.88 ± 41.99	548.17 ± 41.24
Concreteness	402.93 ± 51.94	342.05 ± 48.65	356.32 ± 49.45
Imageability	426.40 ± 95.41	427.97 ± 53.26	427.14 ± 51.88
Word length (number of letters)	4.92 ± 1.03	5.36 ± 1.16	5.32 ± 1.36
Word length (number of syllables)	1.43 ± 0.50	1.51 ± 0.51	1.60 ± 0.49

The range and direction of valence is 1 (extremely unpleasant) to 9 (extremely pleasant).

**Table 3:** 

	Healthy controls (*n* = 17)	Individuals with schizophrenia (*n* = 16)
	Neutral adjectives	
Ample	6.12 ± 1.11	6.87 ± 1.64
Aloof	4.18 ± 0.81	4.20 ± 1.42
Blue*	5.18 ± 1.88	6.75 ± 1.53
Aware	6.47 ± 1.42	6.69 ± 1.62
Blank	4.71 ± 1.10	4.50 ± 1.63
Airy	5.59 ± 1.37	4.94 ± 1.69
Annual	5.29 ± 0.59	5.75 ± 1.24
Basic	5.29 ± 0.69	6.25 ± 1.65
Blond	6.12 ± 1.32	6.38 ± 1.67
Actual	5.29 ± 0.59	5.81 ± 1.52
Broad	5.41 ± 1.18	5.44 ± 1.21
Casual	6.35 ± 1.58	6.69 ± 1.49
Brief	5.59 ± 1.12	5.50 ± 1.59
Brown	5.18 ± 0.88	5.75 ± 1.84
Classic	6.59 ± 1.23	6.94 ± 2.14
Bold	6.13 ± 1.09	6.20 ± 1.61
Common*	5.29 ± 0.85	6.20 ± 1.32
Close	5.76 ± 1.52	5.25 ± 1.48
Civil	6.44 ± 1.31	6.75 ± 1.48
Central*	5.24 ± 0.56	6.13 ± 1.46
Dense	4.65 ± 1.22	4.06 ± 2.11
Constant	5.71 ± 1.05	6.19 ± 1.64
Compact	5.53 ± 1.46	5.63 ± 1.41
Daily*	5.53 ± 1.12	6.75 ± 1.69
Cubic	5.06 ± 0.24	5.20 ± 0.86
Curly	5.47 ± 1.12	6.38 ± 1.67
Equal	6.18 ± 1.70	6.81 ± 1.64
Deep	5.06 ± 1.03	5.33 ± 2.02
Dry	4.71 ± 1.49	4.94 ± 2.32
Direct	6.65 ± 1.54	7.13 ± 1.50
Green	5.94 ± 1.25	6.80 ± 1.90
Long	4.76 ± 1.75	5.75 ± 2.35
Herbal	6.18 ± 1.33	5.88 ± 2.00
Loud	3.71 ± 1.21	3.50 ± 1.75
Large	5.35 ± 0.86	5.44 ± 2.56
Exact	6.31 ± 1.40	7.19 ± 1.56
Full	5.88 ± 1.62	5.73 ± 2.12
Lay	5.65 ± 1.37	5.81 ± 2.40
High	6.06 ± 1.82	6.19 ± 2.29
Flat	4.82 ± 1.07	4.19 ± 1.60
Red	5.24 ± 0.75	5.38 ± 1.63
Mutual	6.35 ± 1.17	6.38 ± 2.16
Plural	5.00 ±0.00	5.63 ± 1.26
Main	5.12 ± 0.33	5.75 ± 1.34
Overt	5.24 ± 0.97	4.50 ± 1.55
Quiet	6.00 ± 1.66	6.06 ± 1.44
Plain	4.88 ± 0.34	4.94 ± 1.24
Raw	4.65 ± 1.11	3.69 ± 1.82
Mild	5.47 ± 0.80	5.75 ± 1.48
Near	5.47 ± 0.87	5.81 ± 1.22
Smooth	6.94 ± 1.25	6.50 ± 1.90
Slim	6.24 ± 1.30	6.81 ± 1.97
White	5.82 ± 2.04	6.44 ± 1.55
Tall	5.94 ± 1.52	7.00 ± 1.86
Tiny	4.53 ± 1.01	5.00 ± 1.59
Yellow	5.65 ± 1.46	5.13 ± 2.36
Wild	5.53 ± 1.01	5.13 ± 1.67
Sharp	5.94 ± 1.68	5.94 ± 2.38
Small	4.94 ± 0.66	5.38 ± 2.06
Thick	4.76 ± 0.56	5.50 ± 1.97

	Positive adjectives	
Calm	7.23 ± 2.02	7.33 ± 1.40
Beautiful	8.29 ± 0.77	7.47 ± 2.13
Adorable	7.82 ± 0.95	7.25 ± 1.88
Clean	7.82 ± 1.07	7.00 ± 2.07
Alive	8.12 ± 1.17	7.93 ± 2.02
Capable	7.18 ± 1.19	6.75 ± 1.95
Brave	7.71 ± 1.31	7.19 ± 1.38
Blessed	8.12 ± 1.11	7.38 ± 2.03
Confident	8.12 ± 0.99	7.75 ± 1.06
Bright	7.71 ± 1.26	7.63 ± 1.31
Erotic	6.71 ± 2.11	6.53 ± 1.64
Elegant	7.94 ± 1.20	7.73 ± 1.39
Famous	6.94 ± 1.52	7.00 ± 1.75
Gentle	7.82 ± 1.19	7.56 ± 1.41
Genial*	6.88 ± 1.54	4.81 ± 1.64
Cute	7.53 ± 1.18	7.40 ± 1.30
Friendly	8.18 ± 0.81	7.94 ± 1.18
Free	7.94 ± 1.25	7.81 ± 2.10
Fabulous	7.94 ± 1.03	7.69 ± 1.25
Funny	8.18 ± 1.01	8.06 ± 1.00
Gifted	7.82 ± 1.01	8.00 ± 1.21
Joyful	8.06 ± 1.25	7.81 ± 1.17
Happy	8.12 ± 1.27	8.00 ± 1.26
Good	7.53 ± 1.01	7.50 ± 1.32
Honest	7.82 ± 0.95	8.13 ± 1.36
Inspired	7.71 ± 1.16	7.56 ± 1.21
Grateful	8.00 ± 1.17	7.63 ± 1.96
Keen	6.41 ± 1.54	6.38 ± 1.67
Hopeful	7.82 ± 1.33	6.75 ± 2.41
Glad	7.35 ± 1.27	7.44 ± 2.03
Loyal	8.00 ± 1.00	7.94 ± 1.24
Magical	7.18 ± 1.19	7.47 ± 1.30
Perfect	7.59 ± 1.06	7.81 ± 2.07
Precious	7.41 ± 1.06	7.53 ± 1.51
Merry	7.94 ± 0.83	7.50 ± 2.03
Lovely	8.06 ± 0.75	7.13 ± 2.36
Lucky	7.53 ± 1.46	7.69 ± 1.49
Kind	7.82 ± 1.38	7.75 ± 1.48
Loved	8.47 ± 0.72	8.07 ± 1.10
Nice	7.82 ± 1.01	7.69 ± 1.40
Protected	7.06 ± 1.98	7.31 ± 1.30
Secure	7.59 ± 1.12	7.75 ± 1.29
Safe	7.6 5± 1.32	7.94 ± 1.29
Right	6.59 ± 1.54	7.25 ± 1.29
Pretty	7.47 ± 1.07	7.31 ± 1.49
Proud	7.00 ± 1.37	7.50 ± 1.41
Romantic	8.12 ± 1.05	7.94 ± 1.34
Satisfied	7.53 ± 1.23	7.25 ± 1.39
Sexy	7.88 ± 1.17	7.25 ± 2.05
Relaxed	7.71 ± 1.10	7.44 ± 1.55
Terrific	7.71 ± 1.10	7.50 ± 1.59
Super	7.59 ± 1.18	7.33 ± 1.59
Special	7.47 ± 1.23	7.25 ± 2.14
Soft	6.47 ± 1.18	6.63 ± 1.86
Tender	6.65 ± 1.32	6.75 ± 1.81
Strong	7.41 ± 1.66	7.00 ± 1.63
Vigorous	6.53 ± 2.10	5.88 ± 1.96
Wise	7.94 ± 1.20	7.63 ± 1.54
Useful	7.24 ± 1.30	7.44 ± 2.06
Wealthy	7.65 ± 1.17	7.63 ± 1.59

	Negative adjectives	
Bloody	1.71 ± 0.92	2.56 ± 2.00
Bored	3.18 ± 1.29	4.00 ± 2.13
Alone	3.82 ± 1.78	3.25 ± 1.61
Clumsy	3.76 ± 1.30	3.44 ± 1.59
Coarse	4.06 ± 1.39	4.44 ± 1.67
Blind	2.35 ± 1.22	2.44 ± 2.06
Abnormal	3.00 ± 1.37	3.50 ± 1.83
Bad	2.29 ± 1.05	3.19 ± 2.17
Angry	2.24 ± 0.90	2.75 ± 1.77
Afraid	2.24 ± 0.90	2.19 ± 1.28
False	3.94 ± 2.36	3.81 ± 1.76
Cruel	1.71 ± 0.77	2.50 ± 2.10
Dirty	2.65 ± 1.58	3.19 ± 1.60
Dull	3.06 ± 1.14	3.25 ± 1.53
Cynic	4.29 ± 1.83	4.06 ± 1.57
Enraged	2.29 ± 1.31	2.63 ± 1.67
Crazy	3.35 ± 1.46	3.25 ± 1.95
Dumb	3.29 ± 1.40	3.00 ± 1.55
Dreadful*	2.00 ± 1.12	3.31 ± 2.09
Dead*	1.29 ± 0.69	2.38 ± 2.03
Ill	2.82 ± 1.78	3.50 ± 2.13
Furious*	2.12 ± 0.78	3.44 ± 2.31
Hostile	2.18 ± 1.19	2.38 ± 1.36
Inferior	2.71 ± 1.26	3.00 ± 1.67
Foolish	3.00 ± 1.22	3.50 ± 1.59
Insane	2.47 ± 1.84	2.50 ± 1.26
Helpless	2.29 ± 1.05	2.94 ± 1.73
Impure	3.94 ± 1.30	3.63 ± 1.67
Guilty*	2.00 ± 0.71	3.00 ± 1.67
Fearful	2.00 ± 1.06	2.44 ± 1.09
Monstrous	3.24 ± 1.60	3.81 ± 2.10
Lost	2.41 ± 0.87	2.56 ± 1.15
Morbid	2.71 ± 1.61	3.19 ± 2.10
Odd	4.00 ± 1.32	3.94 ± 2.08
Lazy	2.94 ± 0.75	3.88 ± 1.93
Malign	3.12 ± 1.36	3.69 ± 1.70
Lonely	2.12 ± 1.27	2.56 ± 1.41
Mad	2.65 ± 1.06	2.81 ± 1.33
Sinful	2.29 ± 1.36	2.94 ± 2.02
Shamed	2.18 ± 1.42	3.38 ± 1.75
Rejected	2.06 ± 1.20	3.00 ± 1.83
Poor	2.47 ± 1.37	2.94 ± 1.53
Selfish	2.00 ± 0.94	2.94 ± 1.34
Sick	1.82 ± 0.95	2.19 ± 1.22
Sad	2.35 ± 0.93	2.93 ± 1.62
Odious	4.12 ± 1.45	4.25 ± 1.57
Scared	2.06 ± 1.20	2.88 ± 2.00
Rude	2.12 ± 0.99	2.75 ± 1.18
Useless	2.24 ± 1.03	2.94 ± 1.53
Terrible	2.00 ± 0.79	2.31 ± 1.20
Unhappy	2.18 ± 0.88	2.69 ± 1.30
Upset	2.35 ± 0.79	2.56 ± 1.31
Weak	2.94 ± 1.30	3.31 ± 1.70
Tough	6.06 ± 1.78	5.38 ± 1.78
Wicked	2.94 ± 1.89	2.88 ± 1.82
Tense	3.12 ± 1.22	4.00 ± 1.93
Ugly	2.35 ± 1.27	3.56 ± 2.10
Stupid	2.47 ± 1.37	2.69 ± 1.40
Terrified	1.76 ± 0.90	2.44 ± 2.13
Wrong	3.12 ± 1.27	2.75 ± 1.13
Violent	1.94 ± 1.56	2.38 ± 1.20
Tragic	1.94 ± 1.09	2.13 ± 1.25
Mean	2.35 ± 1.11	2.63 ± 1.89
Jealous	2.59 ± 1.18	2.69 ± 1.35

*P < .05.

## References

[1] Aleman A, Kahn RS (2005). Strange feelings: do amygdala abnormalities dysregulate the emotional brain in schizophrenia?. *Progress in Neurobiology*.

[2] Edwards J, Jackson HJ, Pattison PE (2002). Emotion recognition via facial expression and affective prosody in schizophrenia: a methodological review. *Clinical Psychology Review*.

[3] Kuperberg GR, Kreher DA, Swain A, Goff DC, Holt DJ (2011). Selective emotional processing deficits to social vignettes in schizophrenia: an ERP study. *Schizophrenia Bulletin*.

[4] Rockstroh B, Junghöfer M, Elbert T, Buodo G, Miller GA (2006). Electromagnetic brain activity evoked by affective stimuli in schizophrenia. *Psychophysiology*.

[5] Phillips LK, Seidman LJ (2008). Emotion processing in persons at risk for schizophrenia. *Schizophrenia Bulletin*.

[6] Green MF, Penn DL, Bentall R (2008). Social cognition in schizophrenia: an NIMH workshop on definitions, assessment, and research opportunities. *Schizophrenia Bulletin*.

[7] Kring AM, Moran EK (2008). Emotional response deficits in schizophrenia: insights from affective science. *Schizophrenia Bulletin*.

[8] Kerr SL, Neale JM (1993). Emotion perception in schizophrenia: specific deficit or further evidence of generalized poor performance?. *Journal of Abnormal Psychology*.

[9] Kring AM, Kerr SL, Smith DA, Neale JM (1993). Flat affect in schizophrenia does not reflect diminished subjective experience of emotion. *Journal of Abnormal Psychology*.

[10] Flack WF, Laird JD, Cavallaro LA (1999). Emotional expression and feeling in schizophrenia: effects of specific expressive behaviors on emotional experiences. *Journal of Clinical Psychology*.

[11] Becerril K, Barch D (2011). Influence of emotional processing on working memory in schizophrenia. *Schizophrenia Bulletin*.

[12] Habel U, Pauly K, Koch K (2010). Emotion-cognition interactions in schizophrenia. *World Journal of Biological Psychiatry*.

[13] Bradley MM, Lang PJ (1994). Measuring emotion: the self-assessment manikin and the semantic differential. *Journal of Behavior Therapy and Experimental Psychiatry*.

[14] Osgood C, Suci G, Tannenbaum P (1957). *The Measurement of Meaning*.

[15] Russell JA (1980). A circumplex model of affect. *Journal of Personality and Social Psychology*.

[16] Dolcos F, Labar KS, Cabeza R (2004). Dissociable effects of arousal and valence on prefrontal activity indexing emotional evaluation and subsequent memory: an event-related fMRI study. *NeuroImage*.

[17] Rozenkrants B, Olofsson JK, Polich J (2008). Affective visual event-related potentials: arousal, valence, and repetition effects for normal and distorted pictures. *International Journal of Psychophysiology*.

[18] Ekman P (1992). An argument for basic emotions. *Cognition and Emotion*.

[19] Sweet LH, Primeau M, Fichtner CG, Lutz G (1998). Dissociation of affect recognition and mood state from blunting in patients with schizophrenia. *Psychiatry Research*.

[20] Hempel RJ, Tulen JH, Van Beveren NJ, Van Steenis HG, Mulder PG, Hengeveld MW (2005). Physiological responsivity to emotional pictures in schizophrenia. *Journal of Psychiatric Research*.

[21] Horan WP, Wynn JK, Kring AM, Simons RF, Green MF (2010). Electrophysiological correlates of emotional responding in schizophrenia. *Journal of Abnormal Psychology*.

[22] Herbener ES, Rosen C, Khine T, Sweeney JA (2007). Failure of positive but not negative emotional valence to enhance memory in schizophrenia. *Journal of Abnormal Psychology*.

[23] Herbener ES, Song W, Khine TT, Sweeney JA (2008). What aspects of emotional functioning are impaired in schizophrenia?. *Schizophrenia Research*.

[24] Quirk SW, Strauss ME (2001). Visual exploration of emotion eliciting images by patients with schizophrenia. *Journal of Nervous and Mental Disease*.

[25] Schneider F, Gur RC, Gur RE, Shtasel DL (1995). Emotional processing in schizophrenia: neurobehavioral probes in relation to psychopathology. *Schizophrenia Research*.

[26] Schneider F, Weiss U, Kessler C (1998). Differential amygdala activation in schizophrenia during sadness. *Schizophrenia Research*.

[27] Takahashi H, Koeda M, Oda K (2004). An fMRI study of differential neural response to affective pictures in schizophrenia. *NeuroImage*.

[28] Cohen AS, Minor KS (2010). Emotional experience in patients with schizophrenia revisited: meta-analysis of laboratory studies. *Schizophrenia Bulletin*.

[29] Heerey EA, Gold JM (2007). Patients with schizophrenia demonstrate dissociation between affective experience and motivated behavior. *Journal of Abnormal Psychology*.

[30] Hempel RJ, Tulen JH, van Beveren NJ, Mulder PG, Hengeveld MW (2007). Subjective and physiological responses to emotion-eliciting pictures in male schizophrenic patients. *International Journal of Psychophysiology*.

[31] Taylor SF, Phan KL, Britton JC, Liberzon I (2005). Neural response to emotional salience in schizophrenia. *Neuropsychopharmacology*.

[32] Holt DJ, Weiss AP, Rauch SL (2005). Sustained activation of the hippocampus in response to fearful faces in schizophrenia. *Biological Psychiatry*.

[33] Rupp CI, Fleischhacker WW, Kemmler G (2005). Olfactory functions and volumetric measures of orbitofrontal and limbic regions in schizophrenia. *Schizophrenia Research*.

[34] Schneider F, Habel U, Reske M, Toni I, Falkai P, Shah NJ (2007). Neural substrates of olfactory processing in schizophrenia patients and their healthy relatives. *Psychiatry Research*.

[35] Burbridge JA, Barch DM (2007). Anhedonia and the experience of emotion in individuals with schizophrenia. *Journal of Abnormal Psychology*.

[36] Dowd EC, Barch DM (2010). Anhedonia and emotional experience in schizophrenia: neural and behavioral indicators. *Biological Psychiatry*.

[37] Strauss GP, Allen DN, Ross SA, Duke LA, Schwartz J (2010). Olfactory hedonic judgment in patients with deficit syndrome schizophrenia. *Schizophrenia Bulletin*.

[38] Strauss GP, Herbener ES (2011). Patterns of emotional experience in schizophrenia: differences in emotional response to visual stimuli are associated with clinical presentation and functional outcome. *Schizophrenia Research*.

[39] Aminoff SR, Jensen J, Lagerberg TV, Andreassen OA, Melle I (2011). Decreased self-reported arousal in schizophrenia during aversive picture viewing compared to bipolar disorder and healthy controls. *Psychiatry Research*.

[40] Bradley MM, Lang PJ (1999). Affective norms for English words (ANEW): instruction manual and affective ratings.

[41] Bradley MM, Lang PJ (1999). International affective digitized sounds (IADS): stimuli, instruction manual and affective ratings.

[42] Kring AM, Barrett LF, Gard DE (2003). On the broad applicability of the affective circumplex: representations of affective knowledge among schizophrenia patients. *Psychological Science*.

[43] Nestor PG, Kimble MO, O’Donnell BF (1997). Aberrant semantic activation in schizophrenia: a neurophysiological study. *American Journal of Psychiatry*.

[44] Mathalon DH, Roach BJ, Ford JM (2010). Automatic semantic priming abnormalities in schizophrenia. *International Journal of Psychophysiology*.

[45] Rossell SL, Shapleske J, David AS (2000). Direct and indirect semantic priming with neutral and emotional words in schizophrenia: relationship to delusions. *Cognitive Neuropsychiatry*.

[46] Niznikiewicz MA, O’Donnell BF, Nestor PG (1997). ERP assessment of visual and auditory language processing in schizophrenia. *Journal of Abnormal Psychology*.

[47] Kubicki M, McCarley RW, Nestor PG (2003). An fMRI study of semantic processing in men with schizophrenia. *NeuroImage*.

[48] Niznikiewicz M (2008). Future directions for examining semantic memory in schizophrenia spectrum disorders. *Clinical EEG and Neuroscience*.

[49] Niznikiewicz M, Mittal MS, Nestor PG, McCarley RW (2010). Abnormal inhibitory processes in semantic networks in schizophrenia. *International Journal of Psychophysiology*.

[50] Rossell SL, Batty RA (2008). Elucidating semantic disorganisation from a word comprehension task: do patients with schizophrenia and bipolar disorder show differential processing of nouns, verbs and adjectives?. *Schizophrenia Research*.

[51] Bayer M, Sommer W, Schacht A (2011). Emotional words impact the mind but not the body: evidence from pupillary responses. *Psychophysiology*.

[52] Bernat E, Bunce S, Shevrin H (2001). Event-related brain potentials differentiate positive and negative mood adjectives during both supraliminal and subliminal visual processing. *International Journal of Psychophysiology*.

[53] Herbert C, Ethofer T, Anders S (2009). Amygdala activation during reading of emotional adjectives—an advantage for pleasant content. *Social Cognitive & Affective Neuroscience*.

[54] Herbert C, Junghofer M, Kissler J (2008). Event related potentials to emotional adjectives during reading. *Psychophysiology*.

[55] Herbert C, Kissler J, Junghöfer M, Peyk P, Rockstroh B (2006). Processing of emotional adjectives: evidence from startle EMG and ERPs. *Psychophysiology*.

[56] Maddock RJ, Garrett AS, Buonocore MH (2003). Posterior cingulate cortex activation by emotional words: fMRI evidence from a valence decision task. *Human Brain Mapping*.

[57] Kissler J, Herbert C, Winkler I, Junghofer M (2009). Emotion and attention in visual word processing: an ERP study. *Biological Psychology*.

[58] Kissler J, Herbert C, Peyk P, Junghofer M (2007). Buzzwords: early cortical responses to emotional words during reading: research report. *Psychological Science*.

[59] Rossell SL (2004). Affective semantic priming in patients with schizophrenia. *Psychiatry Research*.

[60] Suslow T, Roestel C, Droste T, Arolt V (2003). Automatic processing of verbal emotion stimuli in schizophrenia. *Psychiatry Research*.

[61] Klumpp H, Keller J, Miller GA, Casas BR, Best JL, Deldin PJ (2010). Semantic processing of emotional words in depression and schizophrenia. *International Journal of Psychophysiology*.

[62] Harvey PO, Lee J, Horan WP, Ochsner K, Green MF (2011). Do patients with schizophrenia benefit from a self-referential memory bias?. *Schizophrenia Research*.

[63] Hoffman RE, Varanko M, Gilmore J, Mishara AL (2008). Experiential features used by patients with schizophrenia to differentiate ‘voices’ from ordinary verbal thought. *Psychological Medicine*.

[64] Laroi F (2012). How do auditory verbal hallucinations in patients differ from those in non-patients?. *Frontiers in Human Neuroscience*.

[65] Nayani TH, David AS (1996). The auditory hallucination: a phenomenological survey. *Psychological Medicine*.

[66] Lang PJ, Bradley MM, Cuthbert BN (1999). Technical manual and affective ratings. *International Affective Picture System (IAPS)*.

[67] Schneider F, Gur RC, Koch K (2006). Impairment in the specificity of emotion processing in schizophrenia. *American Journal of Psychiatry*.

[68] Seiferth NY, Pauly K, Kellermann T (2009). Neuronal correlates of facial emotion discrimination in early onset schizophrenia. *Neuropsychopharmacology*.

[69] First MB, Spitzer RL, Gibbon M, Williams JBM (1995). *Structured Clinical Interview for DSM-IV Axis II Personality Disorders (SCID-II, Version 2.0)*.

[70] First MB, Spitzer RL, Gibbon M, Williams JBM (2002). *Structured Clinical Interview for DSM-IV Axis I Diagnosis-Patient Edition (SCID-I/P, Version 2.0)*.

[71] Wechsler D (1997). *Wechsler Adult Intelligence Scale: Administration and Scoring Manual*.

[72] Andreasen NC, Endicott J, Spitzer RL, Winokur G (1977). The family history method using diagnostic criteria: reliability and validity. *Archives of General Psychiatry*.

[73] Faraone SV, Seidman LJ, Kremen WS, Pepple JR, Lyons MJ, Tsuang MT (1995). Neuropsychological functioning among the nonpsychotic relatives of schizophrenic patients: a diagnostic efficiency analysis. *Journal of Abnormal Psychology*.

[74] Allen PP, Johns LC, Fu CH, Broome MR, Vythelingum GN, McGuire PK (2004). Misattribution of external speech in patients with hallucinations and delusions. *Schizophrenia Research*.

[75] Johns LC, Rossell S, Frith C (2001). Verbal self-monitoring and auditory verbal hallucinations in patients with schizophrenia. *Psychological Medicine*.

[76] Kucera H, Francis WN (1967). *Computational Analysis of Present-Day American English*.

[77] Kring AM, Gordon AH (1997). Sex differences in emotion: expression, experience, and physiology. *Journal of Personality and Social Psychology*.

[78] Nasser EH, Walders N, Jenkins JH (2002). The experience of schizophrenia: what’s gender got to do with it? A critical review of the current status of research on schizophrenia. *Schizophrenia Bulletin*.

